# Diversity of the Antimicrobial Peptide Genes in Collembola

**DOI:** 10.3390/insects14030215

**Published:** 2023-02-21

**Authors:** Goma Pradhan, Patamarerk Engsontia

**Affiliations:** 1 Division of Biological Science, Faculty of Science, Prince of Songkla University, Hat Yai, Songkhla 90110, Thailand; 2 Molecular Evolution and Computational Biology Research Unit, Faculty of Science, Prince of Songkla University, Hat Yai, Songkhla 90110, Thailand

**Keywords:** antimicrobial peptide, AMP evolution, AMP gene identification, collembola immunity, drug discovery

## Abstract

**Simple Summary:**

Collembola (springtails) are tiny non-insect hexapods living in soil and have an important ecological role as detritivores. How they can survive in the microbe-rich environment for millions of years is unclear. This study used homology-based gene identification methods to identify antimicrobial peptide genes from collembola genomes and transcriptomes. We analyzed five collembola species representing three main suborders and identified 45 antimicrobial peptide genes from five families: diapausin, Alo, diptericin, defensin, and cecropin. These peptides potentially have broad activity against bacteria, fungi, and viruses. This study highlights collembola as a new source for discovering novel AMPs that may help solve the current multidrug-resistant pathogen crisis.

**Abstract:**

Multidrug-resistant bacteria are a current health crisis threatening the world’s population, and scientists are looking for new drugs to combat them. Antimicrobial peptides (AMPs), which are part of the organism’s innate immune system, are a promising new drug class as they can disrupt bacterial cell membranes. This study explored antimicrobial peptide genes in collembola, a non-insect hexapod lineage that has survived in microbe-rich habitats for millions of years, and their antimicrobial peptides have not been thoroughly investigated. We used in silico analysis (homology-based gene identification, physicochemical and antimicrobial property prediction) to identify AMP genes from the genomes and transcriptomes of five collembola representing three main suborders: Entomobryomorpha (*Orchesella cincta*, *Sinella curviseta*), Poduromorpha (*Holacanthella duospinosa*, *Anurida maritima*), and Symphypleona (*Sminthurus viridis*). We identified 45 genes belonging to five AMP families, including (a) cysteine-rich peptides: diapausin, defensin, and Alo; (b) linear α-helical peptide without cysteine: cecropin; (c) glycine-rich peptide: diptericin. Frequent gene gains and losses were observed in their evolution. Based on the functions of their orthologs in insects, these AMPs potentially have broad activity against bacteria, fungi, and viruses. This study provides candidate collembolan AMPs for further functional analysis that could lead to medicinal use.

## 1. Introduction

The emergence of multidrug-resistant bacteria is currently one of the most acute health crises of the 21st century. In 2019, more than one million deaths globally were estimated to be directly associated with drug-resistant bacteria [1], and the number is predicted to reach 10 million deaths annually by 2050 [2]. It is, thus, an urgent issue that requires global action to promote the correct use of antibiotics in humans and farm animals and to find new therapeutic methods, e.g., phage therapy and new drugs, that can effectively kill bacteria [3].

Antimicrobial peptides (AMPs) are promising new drugs for multidrug-resistant bacteria. They are small polypeptide molecules (12–50 amino acids) that are part of the innate immune system of all living things [4,5,6,7]. They are diverse in form and function; many have been demonstrated to kill bacteria, viruses, fungi, protozoans, and nematodes [7]. The most common mode of action of AMPs is disrupting cell membranes, making it difficult for bacteria to develop resistance [8]. AMPs can also inhibit the cell wall, DNA, and protein synthesis of bacteria and disrupt fungi’s mitochondrial membranes [9,10,11]. In addition, AMPs also modulate the host immune response and maintain the host microbiota, which helps control harmful pathogens [12]. More than 40 AMPs are currently in pre-clinical and clinical trials [5].

AMPs from diverse species have been identified and functionally characterized. In the dbAMP database (accessed on 15th December 2022) [13], the total number of curated AMPs are 13,189 peptides. Of these, 10,575 (80%) are from animals, 1772 (13%) are from arthropods, and 704 (5%) are from insects. The insect AMPs were identified mainly from Lepidoptera, Diptera, Coleoptera, and Hymenoptera. The high proportion of insect AMPs suggests that insects are an abundant resource for AMP discovery.

Insect AMPs may be classified into three main categories based on their structures and amino acid compositions [14]: (a) linear α-helical peptides, including cecropins, melittin, moricin, and scarcotoxin that have broad activity against Gram-positive and Gram-negative bacteria, protozoans, viruses, and nematodes [15]; (b) peptides that contain an unusually high proportion of specific amino acids, for example, proline-rich AMP, e.g., drosocin, apidaecin, and pyrrhocoricin, which mainly target Gram-negative bacteria [16], and glycine-rich AMP, e.g., diptericins, gloverins, and attacins, which also have broad activity [17]; (c) cysteine-stabilized AMPs, which form disulfide bridges between conserved cysteine residues, including defensin and defensin-like compounds, e.g., drosomycin, spodoptericin gallerimycin, and Alo, which are mainly active against Gram-positive bacteria and fungi [18,19,20].

Collembola (springtails) are small (0.2 mm–0.6 mm long) non-insect hexapods living in soil and decaying organic matter. They are classified into four taxonomic orders: Poduromorpha, Entomobryomorpha, Symphypleona, and Neeliopleona. Currently, 8700 species have been described [21]. They are detritivores and feed on fungi, bacteria, algae, and actinomycetes, thus playing an essential ecological role in the nutrient cycle [22,23]. Their unique life history challenged by many pathogens suggests that they should possess an effective immune system that allows them to survive in a highly diverse soil microbial community. Indeed, previous studies demonstrated that collembola tolerates some entomopathogenic fungi and bacteria [24,25].

The genetic basis of the immune system to pathogens in collembola is still limited. Most studies focus on responses to abiotic factors, such as heat stress and heavy metals [26,27]. In *O. cincta*, diapausin has been identified and suggested to have a dual role in blocking the uptake of Ca^2+^ channels, aiding collembolan to tolerate heavy metals, and also have antifungal properties [28,29]. Other types of immunity-related genes are also reported, including C-type lectin. In *F. candida*, clusters of genes for penicillin biosynthesis (isopenicillin N synthase and amino adipyl-cysteine-valine synthase) and betalactam synthesis (cephamycin C genes: cmcI and cmcJ) are present in the genome and may explain how they regulate the gut microbiome and internally protect them from soil pathogens [29,30].

Although a few collembola AMPs were identified by automated gene annotation of the genome project [29], a comprehensive comparative study of collembola AMPs genes has not been investigated. In recent years, collembola has gained attention as a model for understanding the terrestrial adaptation of a hexapod lineage and a model for ecotoxicology [27]. New genomes and transcriptomes from collembola, e.g., *F. candida*, *H. duospinosa*, *O. cincta*, and *Si. curviseta,* have become available [27,29,31,32]. The availability of genetic data provides the opportunity to examine the diversity of AMP genes of collembola.

Here, we explored the diversity of AMP genes in the genome and transcriptome of collembola. We used homology-based in silico analysis to identify candidate collembola AMP genes and predict their physicochemical and antimicrobial properties. We identified five AMP families in collembola that reflect potential broad antimicrobial properties. Our study shed light on how collembola adapted to diverse microbial community habitats and discovered new AMPs for future functional study, which may help cope with current drug-resistant pathogen crises.

## 2. Materials and Methods

### 2.1. Collembola Species and Genetic Data

This study investigated AMP genes in five collembola species representing three major collembolan taxonomic orders (Entomobryomorpha: *O. cincta*, *Si. curviseta*; Poduromorpha: *H. duospinosa*, *A. maritima*; Symphypleona: *Sm. viridis*). These species were selected based on the availability of their genetic data. They all have RNA-sequencing reads data, and three of them, *H. duospinosa*, *O. cincta*, and *Si. curviseta*, have the whole-genome data published [16,22,31,32]. For transcriptome analysis, the RNA-sequencing reads were downloaded from the NCBI database. For genome analysis, gene annotations were conducted online using the NCBI-BLAST tool.

### 2.2. Transcriptome Assembly

To construct a transcriptome of each species, downloaded SRA data were de novo assembled using programs equipped in OmicsBox [33]. In brief, the quality of initial reads was examined using FASTQC [34] followed by removing low-quality bases and reads using Trimmomatic [35]. Cleaned reads were then used to assemble a transcriptome using Trinity [36]. The redundant transcripts, which share a sequence similarity higher than 95%, were filtered using the CD-HIT-EST program [37]. The Busco analysis was then used to evaluate the completeness of the transcriptomes [38]. Transcriptome basic statistics were calculated using the Fasta statistics tool in the UseGalaxy server [39].

### 2.3. Identification of AMP Genes

We first downloaded all the reported animal AMP peptide sequences from UniProt databases [40] using the keyword arthropod + antimicrobial peptide, leading to 711 proteins in total (downloaded on 5 February 2021). We then manually filtered out non-AMPs such as large enzymes (e.g., serine protease inhibitors, Toll-like receptors, and lysozymes). This resulted in 698 AMPs used to construct an AMP database for our analysis. We performed gene annotations for each species, one by one. DNA sequences in each collembolan transcriptome were used in a BlastX search (e-value cut-off = 1 × 10^−5^) against the prepared arthropod AMP database using OmicBox. Contigs that have BLAST hits were then grouped according to AMP classes. We manually annotated these hits by retrieving DNA sequences from transcriptomes and translating them using the Expasy translate tool [41]. Correct translation frames were selected based on query sequences of the BLAST results. By the end of these steps, we obtained candidate peptide sequences of AMPs from each species. We refined the search by using these peptides as queries to blast (tBlastn) against other collembola transcriptomes to find additional AMPs that might be missing in the previous steps using the local BLAST program (NCBI-blast-2.12.0+) [42].

As some genes might be missing from the transcriptome due to low or nil expression in the particular tissue/stage, gene annotations were performed to find potential additional genes and complete gene models in three collembola species (*O. cincta*, *Si. curviseta*, and *H. duospinosa*) that have genome data. AMP peptides identified from the transcriptomes were used as queries for the tBlastn search against the whole-genome shotgun (WGS) databases using an online NCBI BLAST (e-value cut-off = 1 × 10^−5^). DNA sequences from blast hits were downloaded and used for predicting gene models using GeneWise [43]. The cDNA and peptide sequences of each AMP gene were reported. Notes on the completeness of the gene models were assigned to gene name as the following suffix: Full = N and C terminus found, NTE = N terminus missing, CTE = C terminus missing, NC = N and C terminus missing.

### 2.4. Physicochemical Property and Functional Prediction

To understand the physicochemical property of candidate AMPs, we first used InterProScan [44] to predict the position of signal peptides and other signature domains. Signal peptides were removed, and only the mature peptide sequences were used to estimate physicochemical properties. We used EMBOSS PEPSTATS [45] to determine sequence length, molecular weight, percentage of polar amino acids, percent positively charged, percent negatively charged, and percent proline and glycine. The isoelectric point and hydrophobic ratio were estimated with the protein report tool found in CLC main workbench V7.9.1 (QIAGEN, Aarhus, Denmark) [46]. The total net charge was calculated with the APD3 [47]. We used Phyre2 to predict the 3D structures of the candidate AMPs based on the most matched peptide [48].

The antimicrobial properties were predicted with SVM (Support Vector Machine), RF (Random Forest), ANN (Artificial Neural Network), and DA (Discriminant Analysis), with algorithmic programs available in Campr3 [49], SVM and RF available in ClassAmp [50], and AB (antibacterial), AV (antiviral), AF (antifungal) prediction available in the iAmpPred tool [51]. The probability scores were reported in the heatmap (probability = 1 is red, and 0 = green). The heatmap diagram was made using Microsoft Excel. We used the DBAASP database [52,53] to predict the antimicrobial activities of collembolan AMPs against specific strains of bacteria and fungi. Three available methods were utilized to predict antibacterial activities against five bacterial strains: *Escherichia coli* ATCC 25922, *Pseudomonas aeruginosa* ATCC 27853, *Klebsiella pneumoniae*, *Staphylococcus aureus* ATC 25923, and *Bacillus subtilis*. The methods included a machine learning (ML) approach based on AMP sequences, a clusterization approach based on AMP sequences, and an ML approach based on AMP sequences and bacterial genomes. To predict antifungal activities against *Candida albicans* and *Saccharomyces cerevisiae*, we used the only available tool, the ML approach based on AMP sequences. For the antimicrobial prediction, active peptides were identified as those with a predicted minimum inhibitory concentration (MIC) less than 25 μg/mL, while non-active peptides were identified as those with a MIC greater than 100 μg/mL. Finally, we used the ML approach based on AMP sequences to predict the toxicity (hemolytic activity) of collembolan AMPs against human erythrocytes. Active peptides were predicted to induce hemolysis greater than 40% at a concentration of less than 40 μg/mL.

### 2.5. Phylogenetic Analysis

**Figure 1:**
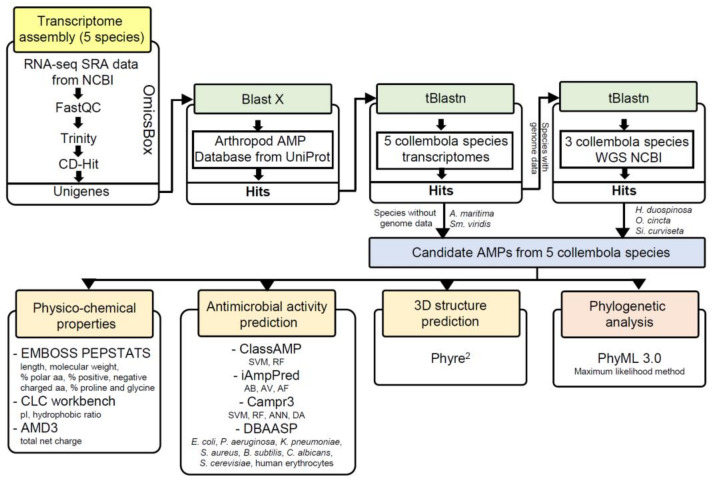
Overview of the AMP gene identification pipeline used in this study. Key steps include transcriptome assembly, AMP genes identification from transcriptomes and genomes, prediction of physicochemical properties, AMP activity, 3D structure, and phylogenetic analysis.

We constructed the phylogenetic relationships of collembola and other arthropod AMPs using the maximum likelihood method. To obtain related AMPs from other arthropods, we performed a BlastP search (e-value cut-off = 1 × 10^−3^) against the arthropod proteins in UniProtKB database using collembola AMPs as queries. Sequences that produce significant blast hits were retrieved and used for phylogenetic analyses (Supplementary Table S1). Peptide sequences of the same AMP family were aligned using MAFFT (E-INS-i) [54]. Gappy regions were removed using Trimal [55]. Maximum likelihood trees were conducted using PhyML [56] and SMS automatic model selection [57]. Branch supports were evaluated using an approximate likelihood ratio test (SH-like). An overview of this work procedure is shown in Figure 1.

## 3. Results

### 3.1. Transcriptome Assembly

Our study constructed five collembola transcriptomes using RNA-sequencing reads from the NCBI database. The number of reads used for the Trinity assembly ranged from ~10 to 52 million reads, in which *H. duospinosa* and *Sm. viridis* have the highest and lowest number of reads, respectively (Table 1). In the final assembly, the number of non-redundant Unigenes varies between ~31,000 and 72,000 unigenes. The average length and N50 value vary from 616 to 1337 base pairs to 907 to 2873 base pairs, respectively. BUSCO analysis, which examines the presence of conserved orthologous genes (Arthropoda_odb9), has a generally high value (88–91.5% in four species and 72% in *O. cincta*), suggesting that the assembled transcriptomes are suitable for gene annotation.

### 3.2. Overview of the Collembolan Candidate AMPs

**Figure 2:**
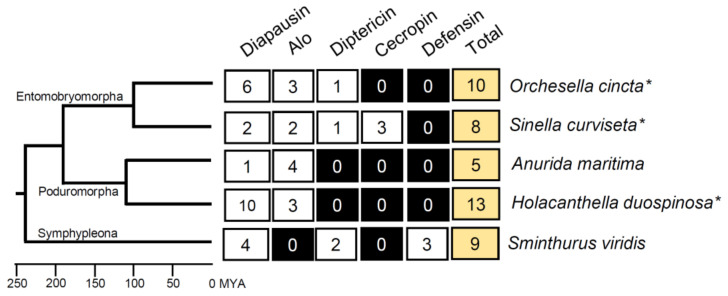
Collembola AMP gene families and the number of genes. Five collembola AMP gene families (diapausin, Alo, diptericin, cecropin, and defensin) are reported in this study. Phylogenetic relationships of five collembola species and the divergent time are inferred from TimeTree.org (Date access: 15 July 2022). Species with genome data are indicated with an asterisk.

Our analysis was analyzed using a homology-based method to identify candidate AMP genes in collembola. All collembola Unigenes from five transcriptomes were searched against a database of various types of >700 arthropod AMPs downloaded from the Uniprot database. All possible orthologous AMP genes in collembola were reported and classified according to the hits. We further searched the genome for species with genome data (*O. cincta*, *Si. curviseta*, and *H. duospinosa*) to find additional genes and complete gene models. We identified only five classes of AMP genes (45 genes in total) in the five collembola species representing three taxonomic orders that span over 250 MY of collembolan evolution (Figure 2). The total number of AMP genes in each species varies between 5 and 13. Diapausin is the only AMP that is present in all five species, whereas Alo is missing in *Sm. viridis* and diptericin is missing in *A. maritima* and *H. duospinosa*. Cecropin and defensin are present only in one species (*Si. curviseta* and *Sm. viridis*, respectively). As genome annotation (*O. cincta*, *Si. curviseta*, and *H. duospinosa*) and transcriptome annotation (*A. maritima* and *Sm. viridis*) yielded similar results, we believe that the number of genes reported here reflect the limited number of direct orthologous genes of known arthropod AMP genes in collembola.

### 3.3. Physicochemical Properties of Collembolan AMPs

To support that the identified collembola AMPs were assigned to the correct AMP families, we investigated whether the mature sequences of collembola AMPs have the same physicochemical properties as their orthologs using in silico prediction tools. These candidate collembolan AMPs are short peptides with an average of 40–97.5 residues in the mature sequences and have a low molecular weight (3930.4–9979.3 Dalton) (Table 2). All of these peptides are cationic at pH 7. The hydrophobic amino acid ratio is low (0.35–0.58), suggesting that these peptides are highly soluble. The percent positive charged amino acid varies between families, with cecropin having the highest value (25.72%). The percent positive charged amino acid is higher than those of negative charged amino acids (10.87–25.72% vs. 2.55–8.57%). The percent proline and glycine also vary between species, while the diptericin has the highest value (26.66%). In general, the physicochemical properties of collembola AMPs are similar to those of their orthologs, particularly the cationic property of all these AMP families and the glycine-rich property of diptericin.

### 3.4. Collembola AMP Family Description

#### 3.4.1. Diapausin Family

**Figure 3:**
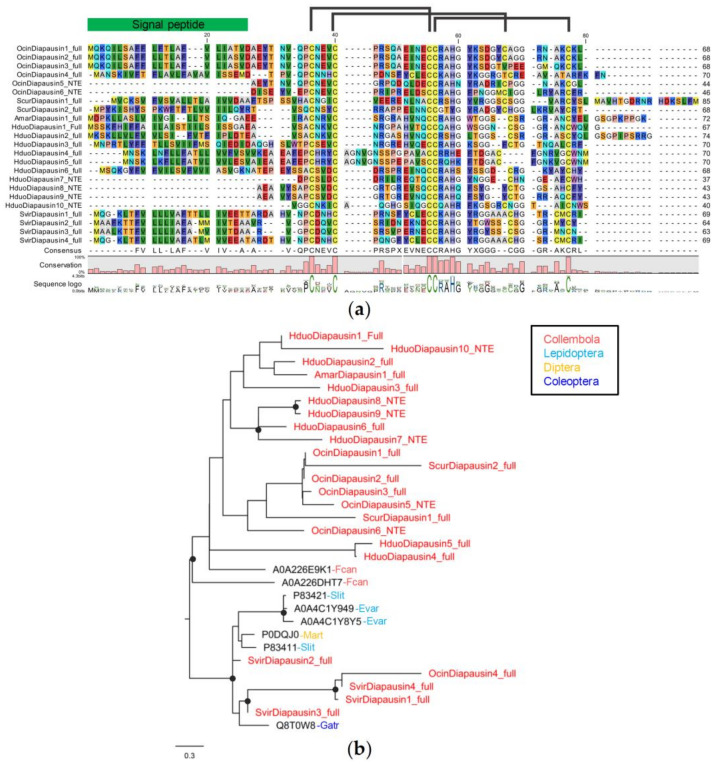
Sequence alignment and phylogenetic relationships of collembola diapausin: (**a**) protein alignment showing disulfide bridges between six conserved cysteine residues; (**b**) Phylogenetic tree of arthropod diapausins. Branch supports (aLRT) higher than 0.9 were indicated with black dots on tree nodes (Abbreviation: Fcan = *F. candida*, Hduo = *H. duospinosa*, Amar = *A. maritima*, Ocin = *O. cincta*, Scur = *Si. curviseta*, Svir = *Sm. viridis*, Slit = *Spodoptera littoralis*, Evar = *Eumeta variegata*, Mart = *Machimus arthriticus*, Gatr = *Gastrophysa atrocyanea*).

Diapausin is the only AMP family present in all five collembola species investigated in this study. We identified 23 diapausin genes from *H. duospinosa* (10 genes), *O. cincta* (six genes), *Sm. viridis* (four genes), *Si. curviseta* (two genes), and *A. maritima* (one gene). Seventeen genes are complete gene models, and six genes are N-terminus-missing. Collembola diapausin are 63–85 amino acids in length and 39–64 amino acids for the mature peptides. Collembola diapausins share six conserved cysteine residues with motif C_1_,X_3_,C_2_,X_9–14_,C_3_C_4_,X_9–11_,C_5_,X_6–9_,C_6_ and potentially have three disulfide bridges between C1 and C3, C2 and C5, and C4 and C6, which are features of insect diapausin (Figure 3a) [58]. Phyre2 predicted collembola diapausins to have two α-helices, and a triple-stranded β-sheet. The predicted 3D structures are most similar to diapausin with antifungal property (Supplementary Table S2).

Most collembola AMPs form a distinct lineage unique to collembola. However, *Sm. viridis* diapausin genes (all four genes) and an *O. cincta* diapausin (OcinDiapausin4) are more closely related to diapausin from other insects (Lepidoptera, Diptera, and Coleoptera) (Figure 3b), suggesting that some collembola diapausins have diverged and evolved independently from other insect diapausins.

#### 3.4.2. Alo Peptide Family

**Figure 4:**
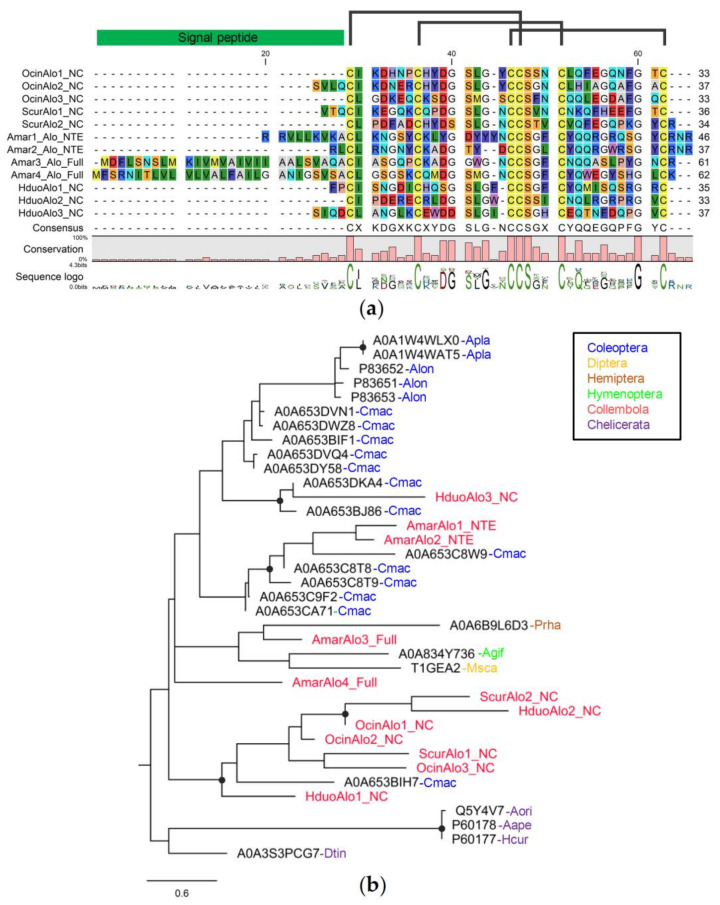
Sequence alignment and phylogenetic relationships of collembola Alo peptides: (**a**) protein alignment showing disulfide bridges between six conserved cysteine residues; (**b**) Phylogenetic tree of arthropod Alo peptides. Branch supports (aLRT) higher than 0.9 were indicated with black dots on tree nodes (Abbreviation: Hduo = *H. duospinosa*, Amar = *A. maritima*, Ocin = *O. cincta*, Scur = *Si. curviseta*, Apla = *Agrilus planipennis*, Alon = *Acrocinus longimanus*, Cmac = *Callosobruchus maculatus*, Prha = *Platymeris rhadamanthus*, Agif = *Aphidius gifuensis*, Msca = *Megaselia scalaris*, Aori = *Agelena orientalis*, Aape = *Agelenopsis aperta*, Hcur = *Hololena curta*, Dtin = *Dinothrombium tinctorium*).

We identified a total of 12 Alo peptides from *A. maritima* (four genes), *H. duospinosa* (three genes), *O. cincta* (three genes), and *Si. curviseta* (two genes). Only two genes from *A. maritima* are full gene models while the rest are N- and C-terminus-missing (8 genes) and N-terminus-missing (two genes). The length of Alo peptides encoded from the full gene models is 61–62 amino acids, and the mature sequences without signal peptides are 34 amino acids. All 12 collembola Alo peptides share six conserved cysteine residues with motif C_1_, X_6_, C_2_, X_7–9_, C_3_C_4_, X_3_, C_5_, X_10_, C_6_, which is a feature of the knottin domain, suggesting three disulfide bridges between C1 and C4, C2 and C5, and C3 and C6 (Figure 4a) [59]. Phyre2 predicted collembola Alo to have a 3D structure most similar to Alo-3 from *A. longimanus*, which exhibits a knottin fold and has an antifungal property (Supplementary Table S2).

Collembola Alo peptides do not form a single monophyletic clade (Figure 4b). Three collembola Alo (AmarAlo1-2, HduoAlo3) are found within clades containing Alo peptides from cowpea weevil, *Callosobruchus maculatus*, (Coleoptera). Seven collembola Alo peptides are more closely related to each other as they form one clade with an Alo gene from *C. maculatus* (Figure 4b). Although *O. cincta*, *Si. curviseta*, *A. maritima*, and *H. duospinosa* have about the same number of Alo genes (2–4 genes), their Alo genes are not direct orthologs (i.e., no 1:1 orthologous relationships), suggesting independent gene gain and loss events in each species rather than the existence of conserved Alo genes in their common ancestor.

#### 3.4.3. Diptericin Family

**Figure 5:**
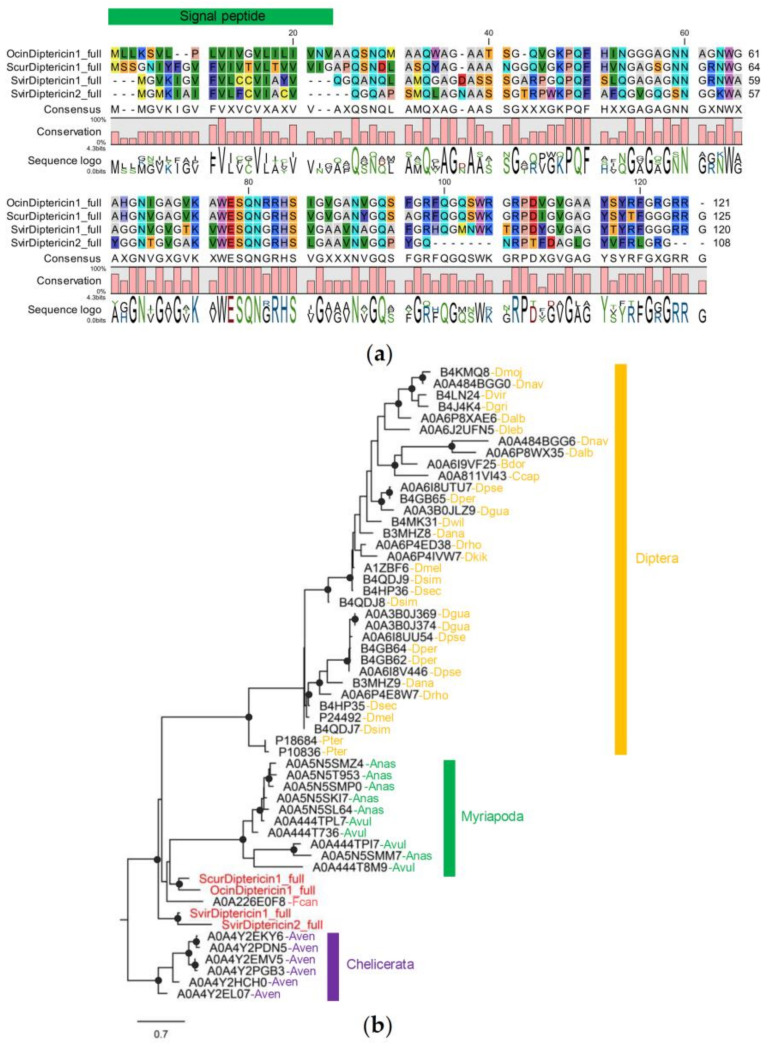
Sequence alignment and phylogenetic relationships of collembola Diptericin: (**a**) protein alignment; (**b**) Phylogenetic tree of arthropod diptericins. Branch supports (aLRT) higher than 0.9 were indicated with black dots on tree nodes (Abbreviation: Ocin = *O. cincta*, Scur = *Si. curviseta*, Svir = *Sm. viridis* Dmoj = *Drosophila mojavensis*, Dnav = *D. navojoa*, Dvir = *D. virilis*, Dgri = *D. grimshawi*, Dalb = *D. albomicans*, Dleb = *D. lebanonensis*, Bdor = *Bactrocera dorsalis*, Ccap = *Ceratitis capitata*, Dpse = *D. pseudoobscura*, Dper = *D. persimilis*, Dgua = *D. guanche*, Dwil = *D. willistoni*, Dana = *D. ananassae*, Drho = *D. rhopaloa*, Dkik = *D. kikkawai*, Dmel = *D. melanogaster*, Dsim = *D. simulans*, Dsec = *D. sechellia*, Pter = *Protophormia terraenovae*, Anas = *Armadillidium nasatum*, Avul = *A. vulgare*, Aven = *Araneus ventricosus*).

We identified a total of four diptericin from *Sm. viridis* (two genes), *O. cincta* (one gene), and *Si. curviseta* (one gene). All of them are full gene models. The lengths of diptericin encoded from the full gene models are 108–125 amino acids and the mature sequences without signal peptides are 89–102 amino acids. These proteins are glycine-rich, constituting 21.35–25.49% glycine and no cysteine in the mature peptides (Figure 5a). Phyre2 cannot predict the structure of collembola diptericin with high confidence, possibly due to the fact that the 3D structure of diptericin has not been characterized, and none exist in the PDB database.

Phylogenetic analysis shows that diptericins of Diptera, Myriapoda, and Chelicerata, but not collembola, form monophyletic clades (Figure 5b). No orthologous genes were found across these main lineages, suggesting that diptericins of the species in the same lineage are the descendants of diptericins found in the common ancestor of each lineage. The branch support values are high throughout the tree, suggesting that the degree of sequence conservation is high; therefore, relationships within and among groups can be inferred.

#### 3.4.4. Cecropin Family

**Figure 6:**
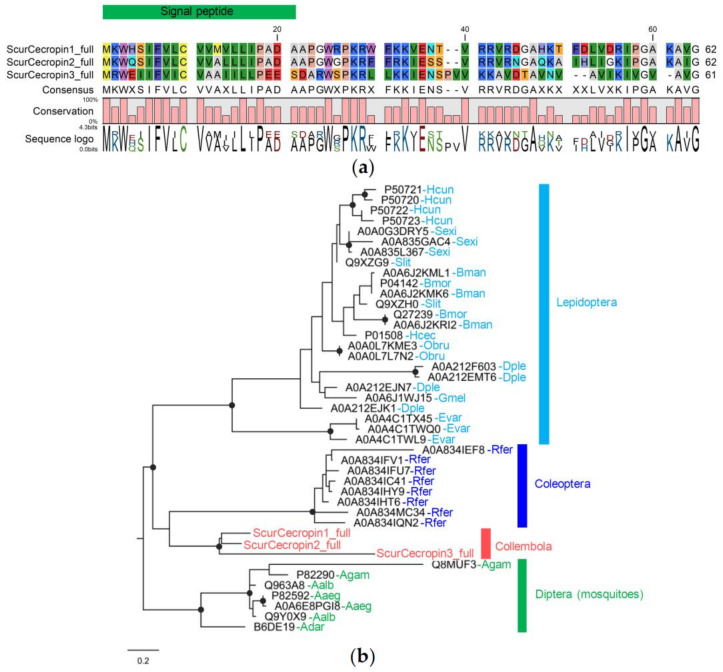
Sequence alignment and phylogenetic relationships of collembola cecropins: (**a**) protein alignment; (**b**) Phylogenetic tree of arthropod cecropins. Branch supports (aLRT) higher than 0.9 were indicated with black dots on tree nodes (Abbreviation: Scur = *Si. curviseta*, Hcun = *Hyphantria cunea*, Sexi = *Spodoptera exigua*, Slit = *Spodoptera litura*, Bman = *Bombyx mandarina*, Bmor = *B. mori*, Hcec = *Hyalophora cecropia*, Obru = *Operophtera brumata*, Dple = *Danaus plexippus*, Gmel = *Galleria mellonella*, Evar = *Eumeta variegata*, Rfer = *Rhynchophorus ferrugineus*, Agam = *Anopheles gambiae*, Adar = *A. darlingi*, Aalb = *Aedes albopictus*, Aaeg = *A. aegypti*).

We identified only three cecropins that are found only in *Si. curviseta*. All of them are full gene models. The lengths of cecropin encoded from the full gene models are 61–62 amino acids, and the mature sequences without signal peptides are 38–41 amino acids. The mature sequences do not have cysteine residues (Figure 6a). This family contains the highest percentage of positively charged amino acids (mean = 25.72), mainly a high frequency of K and R residues. Phyre2 predicted their 3D structure to have two α helices similar to cecropin from the swallowtail butterfly, *Papilio xuthus* (Supplementary Table S2).

Similar to the diptericin family, phylogenetic analysis reveals monophyletic relationships of cecropins from insects in the same lineage, including lepidoptera, coleoptera (red palm weevil), diptera (mosquitoes), and collembola (*Si. curviseta*) (Figure 6b). This suggests that proteins are relatively conserved; thus, the relationship within and among groups can be inferred. Gene family expansion was also observed, particularly eight cecropin genes in *Rhynchophorus ferrugineus*.

#### 3.4.5. Defensin Family

**Figure 7:**
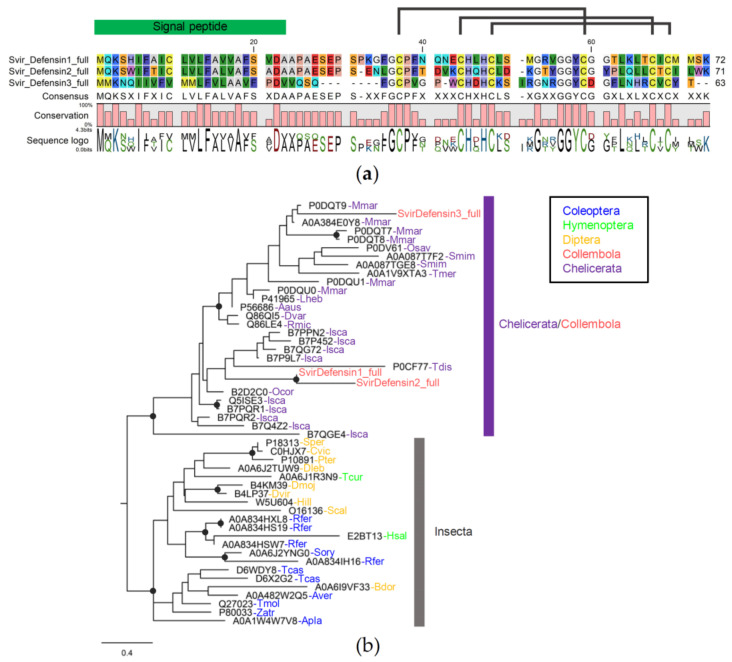
Sequence alignment and phylogenetic relationships of collembola defensin: (**a**) protein alignment showing disulfide bridges between six conserved cysteine residues; (**b**) Phylogenetic tree of arthropod defensins. Branch supports (aLRT) higher than 0.9 were indicated with black dots on tree nodes (Abbreviation: Svir = *Sm. viridis*, Mmar = *Mesobuthus martensii*, Osav = *Ornithodoros savignyi*, Smim = *Stegodyphus mimosarum*, Tmer = *Tropilaelaps mercedesae*, Lheb = *Leiurus hebraeus*, Aaus = *Androctonus australis*, Dvar = *Dermacentor variabilis*, Rmic = *Rhipicephalus microplus*, Isca = *Ixodes scapularis*, Ocor = *Ornithodoros coriaceus*, Tdis = *Tityus discrepans*, Sper = *Sarcophaga peregrina*, Cvic = *Calliphora vicina*, Pter = *Protophormia terraenovae*, Tcur = *Temnothorax curvispinosus*, Dleb = *Drosophila lebanonensis*, Dmoj = *D. mojavensis*, Dvir = *D. virilis*, Hill = *Hermetia illucens*, Scal = *Stomoxys calcitrans*, Rfer = *Rhynchophorus ferrugineus*, Hsal = *Harpegnathos saltator*, Sory = *Sitophilus oryzae*, Tcas = *Tribolium castaneum*, Bdor = *Bactrocera dorsalis*, Aver = *Asbolus verrucosus*, Tmol = *Tenebrio molitor*, Zatr = *Zophobas atratus*, Apla = *Agrilus planipennis*).

We identified only three defensins found only in *Sm. viridis*. All of them are full gene models. The lengths of defensin encoded from the full gene models are 63–72 amino acids, and the mature sequences without signal peptides are 37–49 amino acids. All three defensins share six conserved cysteine residues with motif C_1_, X_5–6_, C_2_, X_3_, C_3_, X_9–10_, C_4_, X_7_, C_5_, X_1_, C_6_, which are the unique structure of the defensin domain, suggesting three disulfide bridges between C1 and C4, C2 and C5, and C3 and C6 (Figure 7a). Phyre2 predicted their 3D structure to have a knottin fold (Supplementary Table S2).

Phylogenetic analysis reveals two main lineages of defensin, one from insects and another from Chelicerata, including three defensins from *Sm. viridis* (Figure 7b). SvirDefensin1 is more closely related to SvirDefensin2 than SvirDefensin3. Defensins in the Chelicerata clade belong to a wide range of taxa, including mites, scorpions, and spiders. In the insect lineage, defensin genes are also from different taxonomic orders, including coleoptera, hymenoptera, and diptera. Of the five collembola species investigated, we can only identify defensins from *Sm. viridis*. As collembola defensins are more closely related to defensins from chelicerata, suggesting its ancient origin, defensin may have been lost in many lineages of collembola rather than the recent gain in *Sm. viridis*.

### 3.5. Functional Prediction

**Figure 8:**
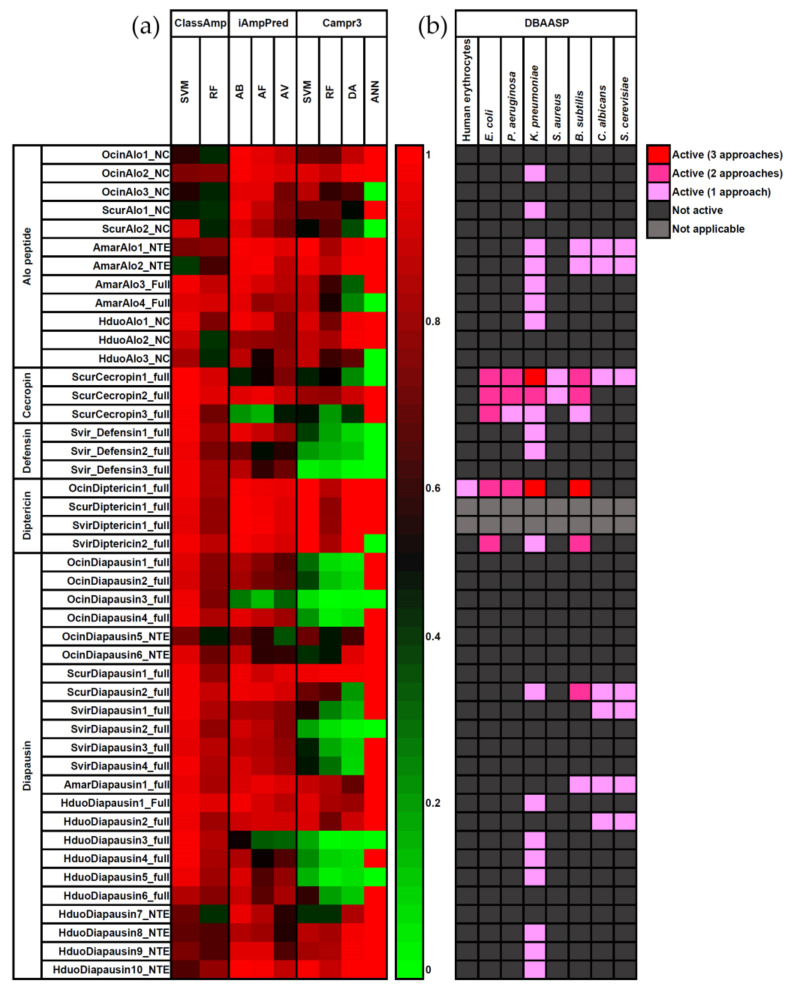
Prediction of antimicrobial activity: (**a**) heat map for the antimicrobial prediction of collembolan AMPs based on three programs (ClassAMP, iAmpPred, and Campr3). The probability value ranges from 0 to 1 and is indicated by the degree of color (SVM = Support Vector Machine, RF = Random Forest, ANN = Artificial Neural Network, DA = Discriminant Analysis, AB = antibacterial property, AV = antiviral property, AF = antifungal property); (**b**) hemolytic activity and strain-specific antimicrobial prediction using DBAASP, where Gram-positive bacteria include *E. coli*, *P. aeruginosa*, and *K. pneumoniae*; Gram-negative bacteria include *S. aureus* and *B. subtilis*; fungi include *C. albicans* and *S. cerevisiae*.

We used three programs (ClassAMP, iAmpPred, and Campr3) to predict the antimicrobial properties of the candidate collembolan AMPs based on sequence properties, such as amino acid composition, family signatures, and physicochemical properties. The results (probability values) were reported as a heatmap (Figure 8a). These programs use different training sets and methods (support vector machine, random forest, artificial neural network, discriminant analysis) and, thus, do not give identical results.

All candidate collembola AMPs were predicted to have antimicrobial properties with high probability scores by at least one program. In general, ClassAmp predicted higher probability values than iAmpPred and Campr3, and the SVM method predicted higher scores than the RF method. The iAmpPred predicted higher probability scores for antibacterial properties than antifungal and antiviral properties. Diptericins have the highest probability scores in all three programs, whereas defensins have the lowest scores, mainly from Campr3. ALOs, cecropins, and diapausins generally have good prediction scores from ClassAMP and iAmpPred, but about 50% of them were predicted with lower probability scores in Campr3. Campr3 (SVM and RF methods) have previously been shown to outperform other tools [49], while ClassAMP may be prone to give false positive results [51,60]. Based on these predictions, diptericin from *O. cincta*, *Sm. viridis,* and *Si. curviseta* has the highest potential for antimicrobial properties. However, we note that the low probability scores predicted by Campr3 can partly be explained by the limitation of collembola AMP sequences in the training set, which limits the prediction power.

Except for OcinDiptericin1, all candidate collembola AMPs were predicted to be non-active against human erythrocytes (Figure 8b), increasing their potential application for medicine. According to the prediction tools in the DBAASP database (Supplementary Table S2), *K. pneumoniae* is sensitive to certain members of all AMP classes, while *B. subtilis* is sensitive to all AMP classes except defensins. *E. coli* and *S. aeruginosa* are susceptible to cecropins and diptericins, whereas *S. aureus* is susceptible to cecropin only. Finally, *C. albicans* and *S. cerevisiae* are sensitive to Alos, cecropins, and diapausins. Among the five AMP classes, cecropins show the most promising activities because all of them (ScurCecropin1-3) are predicted to be active against both Gram-positive and Gram-negative bacteria and ScurCecropin1 is also active against fungi.

## 4. Discussion

### 4.1. Roles of Antimicrobial Peptides in Collembola Immunity

Collembola AMPs identified in this study potentially have broad antimicrobial properties. We believe these peptides were assigned to correct AMP families based on sequence homology and protein family signature (e.g., conserved cysteine residues, physicochemical property, and phylogenetic analysis); thus, their functions may be inferred from other arthropod orthologs. Diapausins, present in all five collembola species, have been reported to have active roles against fungi, e.g., *S. cerevisiae*, *C. albicans*, *C. krusei*, and *Beauveria bassiana* [61,62]. Alo-3 peptide from a beetle, *A. longimanus,* has an active role against fungi *C. albicans* and *C. glabrata* [59]. The strain-specific AMP prediction using the DBAASP database also indicated that two fungi, *C. albicans* and *S. cerevisiae*, are susceptible to Alos and diapausins from collembola. Diptericins from flies were shown to have active roles against Gram-negative bacteria, including *Erwinia herbicola*, *Er. carotovora*, *E. coli*, and *Providencia rettgeri* [63,64,65]. Defensins have broad activity against bacteria, viruses, and fungi but are most effective against Gram-positive bacteria, including *S. aureus* [66]. Insect cecropins can kill both Gram-positive (*Listeria monocytogenes*) and Gram-negative bacteria (*Acinetobacter baumannii* and *P. aeruginosa*), disrupt uropathogenic *E. coli* biofilms, and also exhibit antifungal properties [67,68,69,70]. As functions of collembolan AMP families can only be inferred from their orthologs in other arthropods, further functional analysis is crucial to confirm the prediction.

Our findings have filled the knowledge gaps of how collembola defend themselves against microbes in their natural habitats. Collembolan gut bacteria exhibited antimicrobial properties against various pathogenic bacteria and fungi, thus contributing to the collembola immune system [71]. A cluster of beta-lactam biosynthesis genes producing antibiotics, such as penicillins and cephalosporins, were identified from *F. candida* and other collembolas from different families, but not from the protura, diplura, insects, and other animals, suggesting a single horizontal gene transfer event from bacteria to the common ancestor of collembola [30,72]. Genes in the pathways were upregulated under heat-shock stress and produced beta-lactam products [30]. Although many collembola genomes have been published [27,29,31,32], the AMP families have not been thoroughly investigated and reported. Our analysis shows that collembola have at least five AMP families, potentially contributing to collembolan immunity by having broad activities against bacteria, fungi, and viruses.

### 4.2. Evolution of Collembola AMPs

Our study reveals the dynamic evolution of the collembolan AMP gene families, most importantly, frequent gene gains and losses. Previously, defensin was believed to be the only known ortholog of insect AMP in collembola [20]. We investigated five species of collembola representing three main collembola suborders (Entomobryomorpha, Poduromorpha, Symphypleona) and showed that collembola has at least five AMP families. Diapausins are present in all five collembolan species, suggesting the presence of the diapausin gene in the common ancestor of collembola. Diapausins have been identified in a few insect orders, including coleoptera and lepidoptera; thus, multiple gene gains and losses in different insect lineages may explain the evolution of diapausin in hexapods.

Alo peptides present in four collembolan species from the suborder Entomobryomorpha and Poduromorpha suggest that Alo is another ancient immunity gene of collembola. Previously, Alo peptides present in Hemiptera and Coleoptera were proposed to evolve via horizontal gene transfer from plants or fungi based on the idea that its knottin domain is unique to plants and fungi [20]. However, recent studies show that proteins containing knottin domain play essential roles in many animal toxins, including nettle caterpillars, spiders, scorpions, and cone snails [73]. Thus, the evolution of arthropod Alo peptides may also be explained by multiple gene gains and loss events.

For diptericin, *O. cincta,* and *Si. curviseta*, each has a single gene and *Sm. viridis* has two genes. It seems that diptericin might have been lost in the Poduromorpha lineage, but it has to be confirmed by further analysis that includes more species with complete genomes. Cecropin and defensin are only found in *Si. curviseta* and *Sm. viridis*, respectively. As these two AMPs are widely distributed among insect taxa [20], the origin of these peptides could be dated back to at least the common ancestor of the hexapod. However, these genes might have been lost in many collembola species. Multiple insect AMPs, e.g., drosomycins and thaumatins, have a scattered distribution over insect taxa [20], supporting the fact that gene gain and loss events observed in collembola AMPs are a common phenomenon of hexapod AMPs. We proposed that frequent gene gain and loss in AMP evolution may be due to the broad activities of AMPs, and also, there are many classes of AMP in the genome that can compensate for the function of lost or newly duplicated genes. This process explains the dynamic gene gain and loss events in the evolution of the insect chemoreceptor gene family [74,75]. However, we do not observe a large lineage-specific gene expansion in collembolan AMPs. A recent study on the evolution of Dipteran diptericin suggests trade-offs between having diptericins to combat pathogens and potential risks due to their neuronal toxicity [76]. It might explain the purifying selection against having many copies of genes in the genome. We noted that future analysis on more collembolan species with complete genomes would improve the estimation of gene gain and loss events.

### 4.3. Significance and Implications

Our study supports that collembola are promising new sources for novel AMP discovery. We have identified 45 AMPs from five collembola species that potentially have broad activities against fungi (diapausin, Alo, defensin, and cecropin), bacteria (diptericin, defensin, and cecropin), and viruses (defensin). As the number of AMPs per species is few (5–13 genes), we suspected that collembola might have novel AMPs that are not orthologs of any described AMPs. Future analysis using existing annotation pipelines for novel AMPs [77,78] may reveal the hidden diversity of the collembola AMPs.

Identification of AMPs from unexplored species could lead to numerous candidates, which limit further functional studies when resources are limited. We propose using in silico AMP prediction tools to help choose promising candidates. In our case, we consider Campr3 as the most stringent tool. Therefore, collembolan diptericins, the AMPs that passed all programs (ClassAMP, iAmpPred, and Campr3) with high probability scores, are favorable candidates for further analysis. We note, however, that the novel AMPs distinctly different from the training sets may not be recognized by the AI tools, i.e., give false negative results. Other filtering criteria, such as expression profiles, e.g., AMP genes that show upregulation after organisms were experimentally immunized with pathogens, could be a powerful tool for the screening [79].

Our study serves as a primer for the investigation of collembolan AMPs, which could lead to a better understanding of how collembola can survive in a pathogen-rich environment. It also opens a new road to identifying AMPs that may have a potential use in medicine to help combat the current crisis from multidrug-resistant pathogens.

## Figures and Tables

**Table 1:** Basic information and statistics of the transcriptome assemblies from five collembola.

Taxonomic Order	Species (RNA-Seq SRA ID)	Raw Sequence Reads (Counts)	Unique Contigs (Unigenes)	Contig Length Average (bp)	N50 (bp)	BUSCO Analysis (% Completeness)
Entomobryomorpha	*Orchesella cincta* (SRR935330)	18,994,903	31,396	616	907	72%
	*Sinella curviseta* (SRR7948082)	26,192,990	68,491	1337	2725	91.5%
Poduromorpha	*Anurida maritima* (SRR921564)	12,272,329	36,311	1314	2454	89%
	*Holacanthella duospinosa* (SRR5626546)	52,089,655	72,356	1274	2873	90%
Symphypleona	*Sminthurus viridis* (SRR921641)	10,273,556	48,144	853	1454	88%

**Table 2:** Physicochemical properties of the mature sequence of collembola AMPs by families. Full details for each AMP are reported in Supplementary Table S2.

Properties	Diapausin		Alo Peptide		Diptericin		Cecropin		Defensin	
	Mean	SD	Mean	SD	Mean	SD	Mean	SD	Mean	SD
No. of amino acids	44.48	5.401	35.75	3.596	97.50	5.916	40.00	1.732	44.66	6.658
Molecular weight	4871.0	576.94	3930.4	545.26	9979.3	522.88	4423.2	318.77	4890.3	610.43
pI	8.19	1.345	6.91	1.973	11.1	0.645	11.57	0.607	7.65	1.769
Net charge at pH 7	2.05	2.416	0.44	3.631	7.56	1.546	7.5	1.639	0.92	2.184
Hydrophobic ratio	0.37	0.059	0.35	0.055	0.57	0.012	0.58	0.041	0.44	0.043
% polar amino acids	44.19	4.692	43.09	4.959	40.21	1.509	41.61	4.101	37.36	0.565
% positive charge	16.15	2.843	10.87	5.188	12.01	1.719	25.72	4.219	13.86	4.475
% negative charge	8.33	4.666	8.57	4.805	2.55	0.476	5.82	3.69	7.32	2.711
% proline and glycine	16.07	3.494	15.81	3.514	26.66	1.627	16.52	5.733	20.94	1.941

## Data Availability

The data presented in this study are available in the Supplementary Materials.

## Supplementary Materials

The following supporting information can be downloaded at: https://www.mdpi.com/article/10.3390/insects14030215/s1, Table S1: List of arthropod AMPs UniProt ID used for phylogenetic analyses; Table S2: Collembola AMPs: (a) peptide and DNA sequences, (b) InterProsScan result, (c) Physicochemical property, (d) Phyre2 3D structure prediction, and (4) Predicted antimicrobial properties.

## References

[1] Murray C.J., Ikuta K.S., Sharara F., Swetschinski L., Aguilar G.R., Gray A., Han C., Bisignano C., Rao P., Wool E. (2022). Global Burden of Bacterial Antimicrobial Resistance in 2019: A Systematic Analysis. Lancet.

[2] O’Neill J. (2016). Tackling Drug-Resistant Infections Globally: Final Report and Recommendations.

[3] Talebi Bezmin Abadi A., Rizvanov A.A., Haertlé T., Blatt N.L. (2019). World Health Organization Report: Current Crisis of Antibiotic Resistance. BioNanoScience.

[4] Marr A.K., Gooderham W.J., Hancock R.E. (2006). Antibacterial Peptides for Therapeutic Use: Obstacles and Realistic Outlook. Curr. Opin. Pharmacol..

[5] Browne K., Chakraborty S., Chen R., Willcox M.D., Black D.S., Walsh W.R., Kumar N. (2020). A New Era of Antibiotics: The Clinical Potential of Antimicrobial Peptides. Int. J. Mol. Sci..

[6] Magana M., Pushpanathan M., Santos A.L., Leanse L., Fernandez M., Ioannidis A., Giulianotti M.A., Apidianakis Y., Bradfute S., Ferguson A.L. (2020). The Value of Antimicrobial Peptides in the Age of Resistance. Lancet Infect. Dis..

[7] Rima M., Rima M., Fajloun Z., Sabatier J.-M., Bechinger B., Naas T. (2021). Antimicrobial Peptides: A Potent Alternative to Antibiotics. Antibiotics.

[8] Lazzaro B.P., Zasloff M., Rolff J. (2020). Antimicrobial Peptides: Application Informed by Evolution. Science.

[9] Le C.-F., Fang C.-M., Sekaran S.D. (2017). Intracellular Targeting Mechanisms by Antimicrobial Peptides. Antimicrob. Agents Chemother..

[10] van Eijk E., Wittekoek B., Kuijper E.J., Smits W.K. (2017). DNA Replication Proteins as Potential Targets for Antimicrobials in Drug-Resistant Bacterial Pathogens. J. Antimicrob. Chemother..

[11] Struyfs C., Cammue B.P.A., Thevissen K. (2021). Membrane-Interacting Antifungal Peptides. Front. Cell Dev. Biol..

[12] Hancock R.E.W., Haney E.F., Gill E.E. (2016). The Immunology of Host Defence Peptides: Beyond Antimicrobial Activity. Nat. Rev. Immunol..

[13] Jhong J.-H., Chi Y.-H., Li W.-C., Lin T.-H., Huang K.-Y., Lee T.-Y. (2019). DbAMP: An Integrated Resource for Exploring Antimicrobial Peptides with Functional Activities and Physicochemical Properties on Transcriptome and Proteome Data. Nucleic Acids Res..

[14] Ratcliffe N.A., Mello C.B., Garcia E.S., Butt T.M., Azambuja P. (2011). Insect Natural Products and Processes: New Treatments for Human Disease. Insect Biochem. Mol. Biol..

[15] Brady D., Grapputo A., Romoli O., Sandrelli F. (2019). Insect Cecropins, Antimicrobial Peptides with Potential Therapeutic Applications. Int. J. Mol. Sci..

[16] Cudic M., Condie B.A., Weiner D.J., Lysenko E.S., Xiang Z.Q., Insug O., Bulet P., Otvos L. (2002). Development of Novel Antibacterial Peptides That Kill Resistant Isolates. Peptides.

[17] Buonocore F., Fausto A.M., Della Pelle G., Roncevic T., Gerdol M., Picchietti S. (2021). Attacins: A Promising Class of Insect Antimicrobial Peptides. Antibiotics.

[18] Bulet P., Stöcklin R., Menin L. (2004). Anti-Microbial Peptides: From Invertebrates to Vertebrates. Immunol. Rev..

[19] Silva P.M., Gonçalves S., Santos N.C. (2014). Defensins: Antifungal Lessons from Eukaryotes. Front. Microbiol.

[20] Mylonakis E., Podsiadlowski L., Muhammed M., Vilcinskas A. (2016). Diversity, Evolution and Medical Applications of Insect Antimicrobial Peptides. Philos. Trans. R. Soc. B Biol. Sci..

[21] Cipola N.G., da Silva D.D., Bellini B.C., Hamada N., Thorp J.H., Rogers D.C. (2018). Chapter 2—Class Collembola. Thorp and Covich’s Freshwater Invertebrates.

[22] Faddeeva-Vakhrusheva A., Derks M.F.L., Anvar S.Y., Agamennone V., Suring W., Smit S., van Straalen N.M., Roelofs D. (2016). Gene Family Evolution Reflects Adaptation to Soil Environmental Stressors in the Genome of the Collembolan *Orchesella cincta*. Genome Biol. Evol..

[23] Coulibaly S.F.M., Winck B.R., Akpa-Vinceslas M., Mignot L., Legras M., Forey E., Chauvat M. (2019). Functional Assemblages of Collembola Determine Soil Microbial Communities and Associated Functions. Front. Environ. Sci..

[24] Broza M., Pereira R.M., Stimac J.L. (2001). The Nonsusceptibility of Soil Collembola to Insect Pathogens and Their Potential as Scavengers of Microbial Pesticides. Pedobiologia.

[25] Dromph K.M., Vestergaard S. (2002). Pathogenicity and Attractiveness of Entomopathogenic Hyphomycete Fungi to Collembolans. Appl. Soil Ecol..

[26] Roelofs D., Janssens T.K.S., Timmermans M.J.T.N., Nota B., Mariën J., Bochdanovits Z., Ylstra B., Van Straalen N.M. (2009). Adaptive Differences in Gene Expression Associated with Heavy Metal Tolerance in the Soil Arthropod *Orchesella cincta*. Mol. Ecol..

[27] Faddeeva A., Studer R.A., Kraaijeveld K., Sie D., Ylstra B., Mariën J., Camp H.O.D., Datema E., Dunnen J.T.D., van Straalen N.M. (2015). Collembolan Transcriptomes Highlight Molecular Evolution of Hexapods and Provide Clues on the Adaptation to Terrestrial Life. PLoS ONE.

[28] Kouno T., Mizuguchi M., Tanaka H., Yang P., Mori Y., Shinoda H., Unoki K., Aizawa T., Demura M., Suzuki K. (2007). The Structure of a Novel Insect Peptide Explains Its Ca^2+^ Channel Blocking and Antifungal Activities. Biochemistry.

[29] Faddeeva-Vakhrusheva A., Kraaijeveld K., Derks M.F.L., Anvar S.Y., Agamennone V., Suring W., Kampfraath A.A., Ellers J., Le Ngoc G., van Gestel C.A.M. (2017). Coping with Living in the Soil: The Genome of the Parthenogenetic Springtail *Folsomia candida*. BMC Genom..

[30] Suring W., Meusemann K., Blanke A., Mariën J., Schol T., Agamennone V., Faddeeva-Vakhrusheva A., Berg M.P., Brouwer A., The 1KITE Basal Hexapod Consortium (2017). Evolutionary Ecology of Beta-Lactam Gene Clusters in Animals. Mol. Ecol..

[31] Wu C., Jordan M., Newcomb R., Gemmell N., Bank S., Meusemann K., Dearden P., Duncan E., Grosser S., Rutherford K. (2017). Analysis of the Genome of the New Zealand Giant Collembolan (*Holacanthella duospinosa*) Sheds Light on Hexapod Evolution. BMC Genom..

[32] Zhang F., Ding Y., Zhou Q.-S., Wu J., Luo A., Zhu C.-D. (2019). A High-Quality Draft Genome Assembly of *Sinella curviseta*: A Soil Model Organism (Collembola). Genome Biol. Evol..

[33] Biobam: Bioinformatics Solutions. https://www.biobam.com/omicsbox.

[34] FastQC: A Quality Control Tool for High Throughput Sequence Data. http://www.bioinformatics.babraham.ac.uk/projects/fastqc.

[35] Bolger A.M., Lohse M., Usadel B. (2014). Trimmomatic: A Flexible Trimmer for Illumina Sequence Data. Bioinformatics.

[36] Haas B.J., Papanicolaou A., Yassour M., Grabherr M., Blood P.D., Bowden J., Couger M.B., Eccles D., Li B., Lieber M. (2013). De Novo Transcript Sequence Reconstruction from RNA-Seq: Reference Generation and Analysis with Trinity. Nat. Protoc..

[37] Li W., Godzik A. (2006). Cd-Hit: A Fast Program for Clustering and Comparing Large Sets of Protein or Nucleotide Sequences. Bioinformatics.

[38] Simão F.A., Waterhouse R.M., Ioannidis P., Kriventseva E.V., Zdobnov E.M. (2015). BUSCO: Assessing Genome Assembly and Annotation Completeness with Single-Copy Orthologs. Bioinformatics.

[39] Jalili V., Afgan E., Gu Q., Clements D., Blankenberg D., Goecks J., Taylor J., Nekrutenko A. (2020). The Galaxy Platform for Accessible, Reproducible and Collaborative Biomedical Analyses: 2020 Update. Nucleic Acids Res..

[40] The UniProt Consortium (2023). UniProt: The Universal Protein Knowledgebase in 2023. Nucleic Acids Res..

[41] Gasteiger E., Gattiker A., Hoogland C., Ivanyi I., Appel R.D., Bairoch A. (2003). ExPASy: The Proteomics Server for in-Depth Protein Knowledge and Analysis. Nucleic Acids Res..

[42] Camacho C., Coulouris G., Avagyan V., Ma N., Papadopoulos J., Bealer K., Madden T.L. (2009). BLAST+: Architecture and Applications. BMC Bioinform..

[43] Birney E., Clamp M., Durbin R. (2004). GeneWise and Genomewise. Genome Res..

[44] Quevillon E., Silventoinen V., Pillai S., Harte N., Mulder N., Apweiler R., Lopez R. (2005). InterProScan: Protein Domains Identifier. Nucleic Acids Res..

[45] Rice P., Longden I., Bleasby A. (2000). EMBOSS: The European Molecular Biology Open Software Suite. Trends Genet..

[46] QIAGEN CLC Main Workbench: The User-Friendly Solution for Basic Sequencing Analysis. https://digitalinsights.qiagen.com.

[47] Wang G., Li X., Wang Z. (2016). APD3: The Antimicrobial Peptide Database as a Tool for Research and Education. Nucleic Acids Res..

[48] Kelley L.A., Mezulis S., Yates C.M., Wass M.N., Sternberg M.J.E. (2015). The Phyre2 Web Portal for Protein Modeling, Prediction and Analysis. Nat. Protoc..

[49] Waghu F.H., Barai R.S., Gurung P., Idicula-Thomas S. (2016). CAMPR3: A Database on Sequences, Structures and Signatures of Antimicrobial Peptides. Nucleic Acids Res..

[50] Joseph S., Karnik S., Nilawe P., Jayaraman V.K., Idicula-Thomas S. (2012). ClassAMP: A Prediction Tool for Classification of Antimicrobial Peptides. IEEE/ACM Trans. Comput. Biol. Bioinform..

[51] Meher P.K., Sahu T.K., Saini V., Rao A.R. (2017). Predicting Antimicrobial Peptides with Improved Accuracy by Incorporating the Compositional, Physico-Chemical and Structural Features into Chou’s General PseAAC. Sci. Rep..

[52] Vishnepolsky B., Gabrielian A., Rosenthal A., Hurt D.E., Tartakovsky M., Managadze G., Grigolava M., Makhatadze G.I., Pirtskhalava M. (2018). Predictive Model of Linear Antimicrobial Peptides Active against Gram-Negative Bacteria. J. Chem. Inf. Model..

[53] Vishnepolsky B., Grigolava M., Managadze G., Gabrielian A., Rosenthal A., Hurt D.E., Tartakovsky M., Pirtskhalava M. (2022). Comparative Analysis of Machine Learning Algorithms on the Microbial Strain-Specific AMP Prediction. Brief. Bioinform..

[54] Katoh K., Standley D.M. (2013). MAFFT Multiple Sequence Alignment Software Version 7: Improvements in Performance and Usability. Mol. Biol. Evol..

[55] Capella-Gutiérrez S., Silla-Martínez J.M., Gabaldón T. (2009). TrimAl: A Tool for Automated Alignment Trimming in Large-Scale Phylogenetic Analyses. Bioinformatics.

[56] Guindon S., Dufayard J.-F., Lefort V., Anisimova M., Hordijk W., Gascuel O. (2010). New Algorithms and Methods to Estimate Maximum-Likelihood Phylogenies: Assessing the Performance of PhyML 3.0. Syst. Biol..

[57] Lefort V., Longueville J.-E., Gascuel O. (2017). SMS: Smart Model Selection in PhyML. Mol. Biol. Evol..

[58] Tanaka H., Sato K., Saito Y., Yamashita T., Agoh M., Okunishi J., Tachikawa E., Suzuki K. (2003). Insect Diapause-Specific Peptide from the Leaf Beetle Has Consensus with a Putative Iridovirus Peptide. Peptides.

[59] Barbault F., Landon C., Guenneugues M., Meyer J.-P., Schott V., Dimarcq J.-L., Vovelle F. (2003). Solution Structure of Alo-3: A New Knottin-Type Antifungal Peptide from the Insect *Acrocinus longimanus*. Biochemistry.

[60] Rádai Z., Kiss J., Nagy N.A. (2021). Taxonomic Bias in AMP Prediction of Invertebrate Peptides. Sci. Rep..

[61] Souhail Q.A., Hiromasa Y., Rahnamaeian M., Giraldo M.C., Takahashi D., Valent B., Vilcinskas A., Kanost M.R. (2016). Characterization and Regulation of Expression of an Antifungal Peptide from Hemolymph of an Insect, *Manduca sexta*. Dev. Comp. Immunol..

[62] Li M., Al Souhail Q., Veerapandian R., Vediyappan G., Kanost M. (2019). Investigation of an Antifungal Peptide, Diapausin, from *Manduca sexta*. FASEB J..

[63] Keppi E., Pugsley A.P., Lambert J., Wicker C., Dimarcq J.-L., Hoffmann J.A., Hoffmann D. (1989). Mode of Action of Diptericin A, a Bactericidal Peptide Induced in the Hemolymph of *Phormia terranovae* Larvae. Arch. Insect Biochem. Physiol..

[64] Ishikawa M., Kubo T., Natori S. (1992). Purification and Characterization of a Diptericin Homologue from *Sarcophaga peregrina* (Flesh Fly). Biochem. J..

[65] Unckless R.L., Howick V.M., Lazzaro B.P. (2016). Convergent Balancing Selection on an Antimicrobial Peptide in *Drosophila*. Curr. Biol..

[66] Wu Q., Patočka J., Kuča K. (2018). Insect Antimicrobial Peptides, a Mini Review. Toxins.

[67] Mukherjee K., Mraheil M.A., Silva S., Müller D., Cemic F., Hemberger J., Hain T., Vilcinskas A., Chakraborty T. (2011). Anti-Listeria Activities of *Galleria mellonella* Hemolymph Proteins. Appl. Environ. Microbiol..

[68] Jayamani E., Rajamuthiah R., Larkins-Ford J., Fuchs B.B., Conery A.L., Vilcinskas A., Ausubel F.M., Mylonakis E. (2015). Insect-Derived Cecropins Display Activity against *Acinetobacter baumannii* in a Whole-Animal High-Throughput *Caenorhabditis elegans* Model. Antimicrob. Agents Chemother..

[69] Romoli O., Mukherjee S., Mohid S.A., Dutta A., Montali A., Franzolin E., Brady D., Zito F., Bergantino E., Rampazzo C. (2019). Enhanced Silkworm Cecropin B Antimicrobial Activity against *Pseudomonas aeruginosa* from Single Amino Acid Variation. ACS Infect. Dis..

[70] Kalsy M., Tonk M., Hardt M., Dobrindt U., Zdybicka-Barabas A., Cytrynska M., Vilcinskas A., Mukherjee K. (2020). The Insect Antimicrobial Peptide Cecropin A Disrupts Uropathogenic *Escherichia coli* Biofilms. Npj. Biofilms Microbiomes.

[71] Agamennone V., Roelofs D., van Straalen N.M., Janssens T.K. (2018). Antimicrobial Activity in Culturable Gut Microbial Communities of Springtails. J. Appl. Microbiol..

[72] Roelofs D., Timmermans M.J.T.N., Hensbergen P., van Leeuwen H., Koopman J., Faddeeva A., Suring W., de Boer T.E., Mariën J., Boer R. (2013). A Functional Isopenicillin N Synthase in an Animal Genome. Mol. Biol. Evol..

[73] Mitpuangchon N., Nualcharoen K., Boonrotpong S., Engsontia P. (2021). Identification of Novel Toxin Genes from the Stinging Nettle Caterpillar *Parasa lepida* (Cramer, 1799): Insights into the Evolution of Lepidoptera Toxins. Insects.

[74] Engsontia P., Sangket U., Chotigeat W., Satasook C. (2014). Molecular Evolution of the Odorant and Gustatory Receptor Genes in Lepidopteran Insects: Implications for Their Adaptation and Speciation. J. Mol. Evol..

[75] Engsontia P., Sangket U., Robertson H.M., Satasook C. (2015). Diversification of the Ant Odorant Receptor Gene Family and Positive Selection on Candidate Cuticular Hydrocarbon Receptors. BMC Res. Notes.

[76] Hanson M.A., Lemaitre B., Unckless R.L. (2019). Dynamic Evolution of Antimicrobial Peptides Underscores Trade-Offs Between Immunity and Ecological Fitness. Front. Immunol..

[77] Yoo W.G., Lee J.H., Shin Y., Shim J.-Y., Jung M., Kang B.-C., Oh J., Seong J., Lee H.K., Kong H.S. (2014). Antimicrobial Peptides in the Centipede *Scolopendra subspinipes mutilans*. Funct. Integr. Genom..

[78] Lee J.H., Chung H., Shin Y.P., Kim M.-A., Natarajan S., Veerappan K., Kim S.H., Park J., Hwang J.S. (2020). Deciphering Novel Antimicrobial Peptides from the Transcriptome of *Papilio xuthus*. Insects.

[79] Lee J.H., Chung H., Shin Y.P., Kim M.-A., Natarajan S., Veerappan K., Kim S.H., Park J., Hwang J.S. (2021). Uncovering Antimicrobial Peptide from *Zophobas atratus* Using Transcriptome Analysis. Int. J. Pept. Res. Ther..

